# 2-Amino-4-(4-bromo­phen­yl)-6-meth­oxy-4*H*-benzo[*h*]chromene-3-carbonitrile

**DOI:** 10.1107/S1600536813005461

**Published:** 2013-03-02

**Authors:** Al-anood M. Al-dies, Ahmed M. El-Agrody, Mohamed A. Al-Omar, Abd El-Galil E. Amr, Seik Weng Ng, Edward R. T. Tiekink

**Affiliations:** a Chemistry Department, Faculty of Science, King Khalid University, Abha 61413, PO Box 9004, Saudi Arabia; b Chemistry Department, Faculty of Science, Al-Azhar University, Nasr City, Cairo, 11884, Egypt; c Pharmaceutical Chemistry Department, College of Pharmacy, King Saud University, Riyadh 11451, Saudi Arabia; d Drug Exploration & Development Chair (DEDC), College of Pharmacy, King Saud University, Riyadh 11451, Saudi Arabia; e Applied Organic Chemistry Department, National Research Center, Dokki 12622, Cairo, Egypt; f Department of Chemistry, University of Malaya, 50603 Kuala Lumpur, Malaysia; g Chemistry Department, Faculty of Science, King Abdulaziz University, PO Box 80203 Jeddah, Saudi Arabia

## Abstract

In the title compound, C_21_H_15_BrN_2_O_2_, the 14 non-H atoms of the 4*H*-benzo[*h*]chromene fused-ring system are approximately coplanar (r.m.s. deviation = 0.129 Å). Within this system, the 4*H*-pyran ring adopts a flattened half-chair conformation with the methine C atom lying 0.281 (4) Å above the plane of the remaining atoms (r.m.s. deviation = 0.0446 Å). The bromo­benzene ring is almost perpendicular to the fused-ring system [dihedral angle = 85.34 (13)°]. In the crystal, supra­molecular layers parallel to (101) are sustained by amine–cyano N—H⋯N and amine–meth­oxy N—H⋯O hydrogen bonds. The layers stack with inter­actions of the type (bromo­benzene)C—H⋯π(outer-C_6_ ring of the fused-ring system) connecting them.

## Related literature

For background to biologically active mol­ecules having the 4*H*-chromene or 4*H*-benzochromene residue, see: Sabry *et al.* (2011 ▶); Amin *et al.* (2010 ▶); Kidwai *et al.* (2010 ▶); Singh *et al.* (2010 ▶), For the structure of the fluoro derivative, see: Al-Dies *et al.* (2012 ▶).
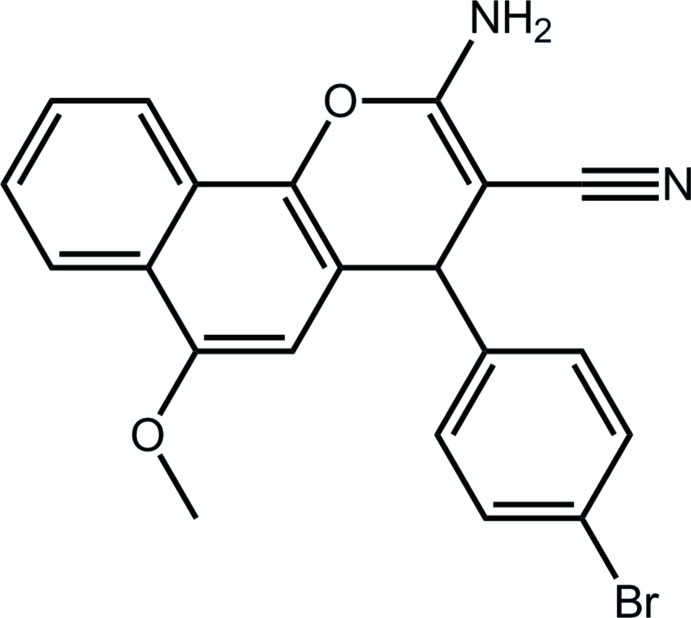


## Experimental

### 

#### Crystal data

C_21_H_15_BrN_2_O_2_
*M* *_r_* = 407.26
Monoclinic, 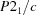

*a* = 6.0823 (7) Å
*b* = 16.6918 (18) Å
*c* = 17.7700 (16) Å
β = 93.646 (9)°
*V* = 1800.4 (3) Å^3^
*Z* = 4
Mo *K*α radiation
μ = 2.30 mm^−1^
*T* = 295 K
0.30 × 0.10 × 0.10 mm

#### Data collection

Agilent SuperNova Dual diffractometer with an Atlas detector
Absorption correction: multi-scan (*CrysAlis PRO*; Agilent, 2011 ▶) *T*_min_ = 0.694, *T*_max_ = 1.000
8976 measured reflections
4144 independent reflections
2250 reflections with *I* > 2σ(*I*)
*R*_int_ = 0.041

#### Refinement

*R*[*F* ^2^ > 2σ(*F* ^2^)] = 0.053
*wR*(*F* ^2^) = 0.150
*S* = 1.03
4144 reflections
243 parameters
2 restraints
H atoms treated by a mixture of independent and constrained refinement
Δρ_max_ = 0.71 e Å^−3^
Δρ_min_ = −0.78 e Å^−3^

### 

Data collection: *CrysAlis PRO* (Agilent, 2011 ▶); cell refinement: *CrysAlis PRO*; data reduction: *CrysAlis PRO*; program(s) used to solve structure: *SHELXS97* (Sheldrick, 2008 ▶); program(s) used to refine structure: *SHELXL97* (Sheldrick, 2008 ▶); molecular graphics: *ORTEP-3 for Windows* (Farrugia, 2012 ▶) and *DIAMOND* (Brandenburg, 2006 ▶); software used to prepare material for publication: *publCIF* (Westrip, 2010 ▶).

## Supplementary Material

Crystal structure: contains datablock(s) global, I. DOI: 10.1107/S1600536813005461/hb7048sup1.cif

Structure factors: contains datablock(s) I. DOI: 10.1107/S1600536813005461/hb7048Isup2.hkl

Supplementary material file. DOI: 10.1107/S1600536813005461/hb7048Isup3.cml

Additional supplementary materials:  crystallographic information; 3D view; checkCIF report

## Figures and Tables

**Table 1:** Hydrogen-bond geometry (Å, °) *Cg*1 is the centroid of the C2–C7 ring.

*D*—H⋯*A*	*D*—H	H⋯*A*	*D*⋯*A*	*D*—H⋯*A*
N1—H1⋯N2^i^	0.88 (1)	2.22 (2)	3.059 (5)	159 (4)
N1—H2⋯O2^ii^	0.87 (3)	2.56 (5)	3.324 (4)	147 (4)
C18—H18⋯*Cg*1^iii^	0.93	2.87	3.528 (4)	129

Symmetry codes: (i) 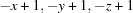; (ii) 
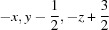
; (iii) 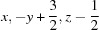.

**Fig. 1.**
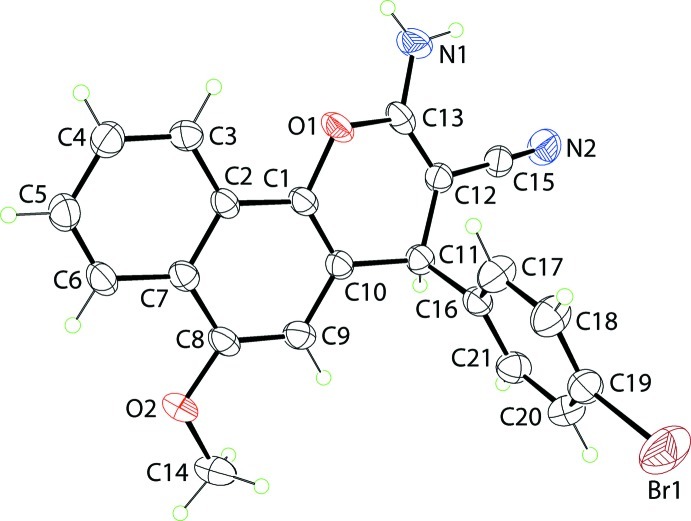
The molecular structure of (I) showing displacement ellipsoids at the 35% probability level.

**Fig. 2.**
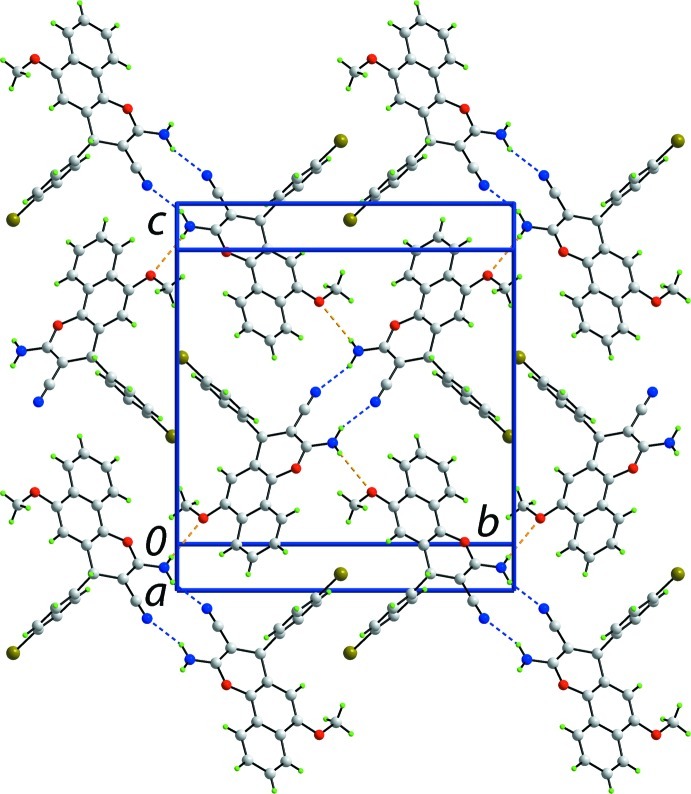
A view of the supramolecular layer parallel to (1 0 1) in (I). The N—H···N and N—H···O hydrogen bonds are shown as blue and orange dashed lines, respectively.

**Fig. 3.**
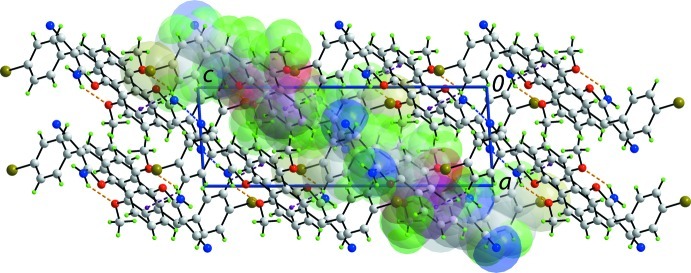
View in projection down the b axis of the crystal packing in (I). The N—H···N, N—H···O and C—H···π interactions are shown as blue, orange and purple dashed lines, respectively. One layer has been highlighted in space-filling mode.

## supplementary crystallographic information

### Comment

Nitrogen-containing heterocyclic compounds having a 4*H*-chromene or 4*H*-benzochromene residue are often found in biologically active molecules (Amin *et al.*, 2010; Kidwai *et al.*, 2010; Singh *et al.*, 2010). As part of our on-going program investigating the chemistry of 4*H*-pyran derivatives (Sabry *et al.*, 2011), we now report the synthesis of the title compound, (I), and its crystal structure.

In (I), Fig. 1, the 4*H*-pyran ring approximates a half-chair conformation with the methine-C11 atom lying 0.281 (4) Å out of the least-squares plane defined by the remaining atoms (O1,C1,C10,C12,C13) which has a r.m.s. deviation of 0.0446 Å. Nevertheless, the 14 non-hydrogen atoms comprising the sequence of three six-membered rings of the 4*H*-benzo[*h*]chromene residue approximate a plane with the r.m.s. deviation of the fitted atoms being 0.129 Å. The bromobenzene ring forms a dihedral angle of 85.34 (13)° with this plane, indicating an almost perpendicular relationship. The methoxy group is co-planar with the ring to which it is connected as seen in the value of the C14—O2—C8—C7 torsion angle of 177.4 (3)°. The overall structure resembles closely the recently reported fluoro derivative (Al-Dies *et al.*, 2012).

In the crystal packing, amine-*N*—*H*···*N*(cyano) hydrogen bonds between centrosymmetrically related molecules lead to dimeric aggregates, stabilized by 12-membered {···HNC_3_N}_2_ synthons, which are connected into a supramolecular layer parallel to (1 0 1) by amine-*N*—*H*···*O*(methoxy) hydrogen bonds, Fig. 2 and Table 1. Layers stack with the main interactions between them being of the type (bromobenzene)C—H···π(outer-C_6_ ring of the fused ring system), Fig. 3 and Table 1.

### Experimental

A solution of 4-methoxy-1-naphthol (0.01 mol) in EtOH (30 ml) was treated with α-cyano-*p*-bromocinnamonitrile (0.01 mol) and piperidine (0.5 ml). The reaction mixture was heated until complete precipitation occurred (reaction time: 60 min). The solid product which formed was collected by filtration and recrystallized from ethanol to give the title compound as yellow prisms; *M*. pt: 503–504 K.

### Refinement

The C-bound H atoms were geometrically placed (C—H = 0.93–0.98 Å) and refined as riding with *U_iso_*(H) = 1.2–1.5*U_eq_*(C). The N-bound H atoms were refined with the distance restraint N—H = 0.88±0.01 Å and with free *U*_eq_.

### Crystal data

**Table:**

C_21_H_15_BrN_2_O_2_	*F*(000) = 824
*M**_r_* = 407.26	*D*_x_ = 1.502 Mg m^−^^3^
Monoclinic, *P*2_1_/*c*	Mo *K*α radiation, λ = 0.71073 Å
Hall symbol: -P 2ybc	Cell parameters from 1634 reflections
*a* = 6.0823 (7) Å	θ = 3.4–27.5°
*b* = 16.6918 (18) Å	µ = 2.30 mm^−^^1^
*c* = 17.7700 (16) Å	*T* = 295 K
β = 93.646 (9)°	Prism, yellow
*V* = 1800.4 (3) Å^3^	0.30 × 0.10 × 0.10 mm
*Z* = 4	

### Data collection

**Table:**

Agilent SuperNova Dual diffractometer with an Atlas detector	4144 independent reflections
Radiation source: SuperNova (Mo) X-ray Source	2250 reflections with *I* > 2σ(*I*)
Mirror monochromator	*R*_int_ = 0.041
Detector resolution: 10.4041 pixels mm^-1^	θ_max_ = 27.6°, θ_min_ = 3.4°
ω scan	*h* = −7→7
Absorption correction: multi-scan (*CrysAlis PRO*; Agilent, 2011)	*k* = −15→21
*T*_min_ = 0.694, *T*_max_ = 1.000	*l* = −22→23
8976 measured reflections	

### Refinement

**Table:**

Refinement on *F*^2^	Primary atom site location: structure-invariant direct methods
Least-squares matrix: full	Secondary atom site location: difference Fourier map
*R*[*F*^2^ > 2σ(*F*^2^)] = 0.053	Hydrogen site location: inferred from neighbouring sites
*wR*(*F*^2^) = 0.150	H atoms treated by a mixture of independent and constrained refinement
*S* = 1.03	*w* = 1/[σ^2^(*F*_o_^2^) + (0.057*P*)^2^ + 0.4428*P*] where *P* = (*F*_o_^2^ + 2*F*_c_^2^)/3
4144 reflections	(Δ/σ)_max_ < 0.001
243 parameters	Δρ_max_ = 0.71 e Å^−^^3^
2 restraints	Δρ_min_ = −0.78 e Å^−^^3^

### Special details

**Table:**

Geometry. All s.u.'s (except the s.u. in the dihedral angle between two l.s. planes) are estimated using the full covariance matrix. The cell s.u.'s are taken into account individually in the estimation of s.u.'s in distances, angles and torsion angles; correlations between s.u.'s in cell parameters are only used when they are defined by crystal symmetry. An approximate (isotropic) treatment of cell s.u.'s is used for estimating s.u.'s involving l.s. planes.
Refinement. Refinement of *F*^2^ against ALL reflections. The weighted *R*-factor *wR* and goodness of fit *S* are based on *F*^2^, conventional *R*-factors *R* are based on *F*, with *F* set to zero for negative *F*^2^. The threshold expression of *F*^2^ > 2σ(*F*^2^) is used only for calculating *R*-factors(gt) *etc*. and is not relevant to the choice of reflections for refinement. *R*-factors based on *F*^2^ are statistically about twice as large as those based on *F*, and *R*- factors based on ALL data will be even larger.

### Fractional atomic coordinates and isotropic or equivalent isotropic displacement parameters (Å^2^)

**Table:**

	*x*	*y*	*z*	*U*_iso_*/*U*_eq_	
Br1	0.16537 (9)	0.98612 (4)	0.33868 (3)	0.0969 (3)	
O1	0.0181 (4)	0.64654 (15)	0.64437 (13)	0.0504 (6)	
O2	0.1773 (4)	0.92192 (15)	0.81237 (13)	0.0573 (7)	
N1	0.1307 (6)	0.5382 (2)	0.5848 (2)	0.0582 (8)	
H1	0.207 (7)	0.513 (3)	0.552 (2)	0.093 (17)*	
H2	0.008 (4)	0.517 (3)	0.598 (3)	0.089 (17)*	
N2	0.6286 (6)	0.5888 (2)	0.50299 (19)	0.0676 (9)	
C1	0.0644 (5)	0.7191 (2)	0.68158 (16)	0.0393 (8)	
C2	−0.0802 (5)	0.73721 (19)	0.73914 (16)	0.0375 (7)	
C3	−0.2638 (6)	0.6894 (2)	0.75334 (18)	0.0453 (8)	
H3	−0.2931	0.6437	0.7246	0.054*	
C4	−0.3989 (6)	0.7094 (2)	0.80851 (19)	0.0520 (9)	
H4	−0.5225	0.6783	0.8160	0.062*	
C5	−0.3531 (6)	0.7762 (2)	0.8539 (2)	0.0547 (10)	
H5	−0.4446	0.7887	0.8922	0.066*	
C6	−0.1753 (6)	0.8235 (2)	0.84260 (18)	0.0500 (9)	
H6	−0.1447	0.8672	0.8741	0.060*	
C7	−0.0360 (5)	0.8068 (2)	0.78307 (17)	0.0405 (8)	
C8	0.1447 (6)	0.8565 (2)	0.76620 (17)	0.0430 (8)	
C9	0.2710 (5)	0.8382 (2)	0.70790 (18)	0.0446 (8)	
H9	0.3847	0.8725	0.6964	0.054*	
C10	0.2321 (5)	0.7680 (2)	0.66466 (16)	0.0390 (7)	
C11	0.3774 (5)	0.7476 (2)	0.60075 (17)	0.0411 (8)	
H11	0.5311	0.7556	0.6192	0.049*	
C12	0.3481 (5)	0.6601 (2)	0.58086 (17)	0.0419 (8)	
C13	0.1747 (6)	0.6157 (2)	0.60137 (18)	0.0455 (8)	
C14	0.3613 (7)	0.9720 (2)	0.8004 (2)	0.0635 (11)	
H14A	0.3620	1.0166	0.8345	0.095*	
H14B	0.3509	0.9913	0.7494	0.095*	
H14C	0.4949	0.9419	0.8092	0.095*	
C15	0.5037 (6)	0.6215 (2)	0.53744 (19)	0.0479 (9)	
C16	0.3287 (5)	0.8045 (2)	0.53448 (17)	0.0409 (8)	
C17	0.1278 (6)	0.8030 (3)	0.4940 (2)	0.0577 (10)	
H17	0.0232	0.7651	0.5058	0.069*	
C18	0.0789 (6)	0.8566 (3)	0.4364 (2)	0.0627 (11)	
H18	−0.0580	0.8551	0.4100	0.075*	
C19	0.2331 (7)	0.9121 (2)	0.41810 (19)	0.0549 (10)	
C20	0.4365 (6)	0.9136 (2)	0.4556 (2)	0.0581 (10)	
H20	0.5421	0.9504	0.4423	0.070*	
C21	0.4832 (6)	0.8596 (2)	0.51378 (19)	0.0505 (9)	
H21	0.6214	0.8606	0.5394	0.061*	

### Atomic displacement parameters (Å^2^)

**Table:**

	*U*^11^	*U*^22^	*U*^33^	*U*^12^	*U*^13^	*U*^23^
Br1	0.1155 (5)	0.0841 (4)	0.0888 (4)	−0.0019 (3)	−0.0114 (3)	0.0431 (3)
O1	0.0523 (13)	0.0429 (15)	0.0572 (13)	−0.0032 (11)	0.0139 (11)	−0.0239 (12)
O2	0.0760 (17)	0.0383 (15)	0.0578 (14)	−0.0057 (13)	0.0074 (12)	−0.0170 (12)
N1	0.063 (2)	0.0407 (19)	0.073 (2)	−0.0020 (17)	0.0213 (18)	−0.0202 (17)
N2	0.079 (2)	0.054 (2)	0.073 (2)	0.0104 (19)	0.0324 (18)	0.0012 (18)
C1	0.0484 (19)	0.0320 (18)	0.0372 (16)	0.0036 (15)	0.0016 (15)	−0.0055 (14)
C2	0.0478 (18)	0.0313 (17)	0.0333 (15)	0.0080 (15)	0.0011 (14)	−0.0027 (14)
C3	0.056 (2)	0.0330 (19)	0.0466 (19)	0.0026 (16)	0.0040 (16)	0.0014 (16)
C4	0.056 (2)	0.049 (2)	0.053 (2)	0.0027 (18)	0.0115 (18)	0.0047 (18)
C5	0.066 (2)	0.050 (2)	0.050 (2)	0.008 (2)	0.0177 (18)	−0.0008 (18)
C6	0.070 (2)	0.039 (2)	0.0417 (18)	0.0098 (19)	0.0078 (17)	−0.0074 (16)
C7	0.0516 (19)	0.0335 (18)	0.0361 (16)	0.0076 (16)	−0.0003 (15)	0.0001 (14)
C8	0.057 (2)	0.0304 (18)	0.0404 (17)	0.0094 (16)	−0.0021 (16)	−0.0060 (15)
C9	0.0510 (19)	0.0344 (19)	0.0482 (19)	0.0006 (16)	0.0011 (16)	−0.0017 (15)
C10	0.0486 (18)	0.0333 (18)	0.0353 (16)	0.0043 (16)	0.0041 (14)	−0.0009 (14)
C11	0.0445 (18)	0.040 (2)	0.0390 (17)	0.0029 (16)	0.0023 (14)	−0.0008 (15)
C12	0.0511 (19)	0.0356 (19)	0.0397 (17)	0.0065 (16)	0.0087 (15)	0.0006 (15)
C13	0.056 (2)	0.041 (2)	0.0400 (17)	0.0097 (18)	0.0032 (16)	−0.0114 (16)
C14	0.066 (2)	0.041 (2)	0.083 (3)	−0.005 (2)	0.000 (2)	−0.016 (2)
C15	0.063 (2)	0.037 (2)	0.0444 (18)	0.0100 (18)	0.0124 (17)	0.0034 (16)
C16	0.0457 (18)	0.0371 (19)	0.0399 (17)	0.0018 (16)	0.0032 (15)	−0.0047 (15)
C17	0.052 (2)	0.062 (3)	0.059 (2)	−0.0083 (19)	−0.0018 (18)	0.013 (2)
C18	0.057 (2)	0.071 (3)	0.059 (2)	−0.005 (2)	−0.0058 (19)	0.015 (2)
C19	0.076 (3)	0.043 (2)	0.0458 (19)	0.004 (2)	−0.0022 (19)	0.0060 (17)
C20	0.066 (2)	0.047 (2)	0.062 (2)	−0.014 (2)	0.008 (2)	0.0042 (19)
C21	0.051 (2)	0.047 (2)	0.053 (2)	−0.0032 (18)	−0.0037 (17)	−0.0006 (18)

### Geometric parameters (Å, º)

**Table:**

Br1—C19	1.901 (3)	C8—C9	1.363 (5)
O1—C13	1.360 (4)	C9—C10	1.413 (4)
O1—C1	1.399 (4)	C9—H9	0.9300
O2—C8	1.372 (4)	C10—C11	1.521 (4)
O2—C14	1.423 (5)	C11—C12	1.510 (5)
N1—C13	1.349 (5)	C11—C16	1.528 (4)
N1—H1	0.877 (10)	C11—H11	0.9800
N1—H2	0.874 (10)	C12—C13	1.357 (5)
N2—C15	1.144 (4)	C12—C15	1.414 (5)
C1—C10	1.355 (5)	C14—H14A	0.9600
C1—C2	1.423 (4)	C14—H14B	0.9600
C2—C3	1.408 (5)	C14—H14C	0.9600
C2—C7	1.416 (4)	C16—C17	1.378 (5)
C3—C4	1.360 (5)	C16—C21	1.382 (5)
C3—H3	0.9300	C17—C18	1.378 (5)
C4—C5	1.395 (5)	C17—H17	0.9300
C4—H4	0.9300	C18—C19	1.372 (5)
C5—C6	1.364 (5)	C18—H18	0.9300
C5—H5	0.9300	C19—C20	1.368 (5)
C6—C7	1.424 (5)	C20—C21	1.387 (5)
C6—H6	0.9300	C20—H20	0.9300
C7—C8	1.424 (5)	C21—H21	0.9300
			
C13—O1—C1	117.8 (3)	C12—C11—C16	114.0 (3)
C8—O2—C14	117.6 (3)	C10—C11—C16	110.1 (3)
C13—N1—H1	120 (3)	C12—C11—H11	107.8
C13—N1—H2	120 (3)	C10—C11—H11	107.8
H1—N1—H2	118 (5)	C16—C11—H11	107.8
C10—C1—O1	123.2 (3)	C13—C12—C15	117.1 (3)
C10—C1—C2	122.6 (3)	C13—C12—C11	123.2 (3)
O1—C1—C2	114.1 (3)	C15—C12—C11	119.7 (3)
C3—C2—C7	119.4 (3)	N1—C13—C12	127.7 (3)
C3—C2—C1	122.8 (3)	N1—C13—O1	110.5 (3)
C7—C2—C1	117.8 (3)	C12—C13—O1	121.9 (3)
C4—C3—C2	120.9 (3)	O2—C14—H14A	109.5
C4—C3—H3	119.6	O2—C14—H14B	109.5
C2—C3—H3	119.6	H14A—C14—H14B	109.5
C3—C4—C5	120.3 (4)	O2—C14—H14C	109.5
C3—C4—H4	119.8	H14A—C14—H14C	109.5
C5—C4—H4	119.8	H14B—C14—H14C	109.5
C6—C5—C4	120.6 (3)	N2—C15—C12	178.6 (4)
C6—C5—H5	119.7	C17—C16—C21	117.9 (3)
C4—C5—H5	119.7	C17—C16—C11	120.8 (3)
C5—C6—C7	120.7 (3)	C21—C16—C11	121.2 (3)
C5—C6—H6	119.7	C18—C17—C16	121.2 (4)
C7—C6—H6	119.7	C18—C17—H17	119.4
C2—C7—C8	119.0 (3)	C16—C17—H17	119.4
C2—C7—C6	118.0 (3)	C19—C18—C17	119.8 (3)
C8—C7—C6	123.1 (3)	C19—C18—H18	120.1
C9—C8—O2	124.5 (3)	C17—C18—H18	120.1
C9—C8—C7	120.6 (3)	C20—C19—C18	120.5 (3)
O2—C8—C7	114.9 (3)	C20—C19—Br1	119.7 (3)
C8—C9—C10	121.1 (3)	C18—C19—Br1	119.7 (3)
C8—C9—H9	119.5	C19—C20—C21	119.1 (4)
C10—C9—H9	119.5	C19—C20—H20	120.4
C1—C10—C9	118.8 (3)	C21—C20—H20	120.4
C1—C10—C11	120.8 (3)	C16—C21—C20	121.4 (3)
C9—C10—C11	120.4 (3)	C16—C21—H21	119.3
C12—C11—C10	109.0 (3)	C20—C21—H21	119.3
			
C13—O1—C1—C10	14.6 (4)	C8—C9—C10—C11	−178.8 (3)
C13—O1—C1—C2	−165.3 (3)	C1—C10—C11—C12	−17.0 (4)
C10—C1—C2—C3	175.4 (3)	C9—C10—C11—C12	162.5 (3)
O1—C1—C2—C3	−4.7 (4)	C1—C10—C11—C16	108.7 (3)
C10—C1—C2—C7	−4.1 (4)	C9—C10—C11—C16	−71.8 (4)
O1—C1—C2—C7	175.8 (3)	C10—C11—C12—C13	17.6 (4)
C7—C2—C3—C4	0.1 (5)	C16—C11—C12—C13	−105.8 (3)
C1—C2—C3—C4	−179.4 (3)	C10—C11—C12—C15	−163.8 (3)
C2—C3—C4—C5	−2.3 (5)	C16—C11—C12—C15	72.8 (4)
C3—C4—C5—C6	1.5 (5)	C15—C12—C13—N1	−0.6 (5)
C4—C5—C6—C7	1.6 (5)	C11—C12—C13—N1	178.1 (3)
C3—C2—C7—C8	−177.4 (3)	C15—C12—C13—O1	178.7 (3)
C1—C2—C7—C8	2.1 (4)	C11—C12—C13—O1	−2.7 (5)
C3—C2—C7—C6	2.8 (4)	C1—O1—C13—N1	165.0 (3)
C1—C2—C7—C6	−177.7 (3)	C1—O1—C13—C12	−14.4 (4)
C5—C6—C7—C2	−3.7 (5)	C12—C11—C16—C17	56.2 (4)
C5—C6—C7—C8	176.6 (3)	C10—C11—C16—C17	−66.6 (4)
C14—O2—C8—C9	−2.8 (5)	C12—C11—C16—C21	−124.5 (3)
C14—O2—C8—C7	177.4 (3)	C10—C11—C16—C21	112.6 (4)
C2—C7—C8—C9	1.1 (5)	C21—C16—C17—C18	−2.2 (6)
C6—C7—C8—C9	−179.1 (3)	C11—C16—C17—C18	177.0 (3)
C2—C7—C8—O2	−179.0 (3)	C16—C17—C18—C19	0.7 (6)
C6—C7—C8—O2	0.7 (4)	C17—C18—C19—C20	1.3 (6)
O2—C8—C9—C10	177.5 (3)	C17—C18—C19—Br1	−179.9 (3)
C7—C8—C9—C10	−2.6 (5)	C18—C19—C20—C21	−1.7 (6)
O1—C1—C10—C9	−177.2 (3)	Br1—C19—C20—C21	179.6 (3)
C2—C1—C10—C9	2.7 (5)	C17—C16—C21—C20	1.9 (5)
O1—C1—C10—C11	2.3 (5)	C11—C16—C21—C20	−177.3 (3)
C2—C1—C10—C11	−177.8 (3)	C19—C20—C21—C16	0.0 (6)
C8—C9—C10—C1	0.7 (5)		

### Hydrogen-bond geometry (Å, º)

Cg1 is the centroid of the C2–C7 ring.

**Table:**

*D*—H···*A*	*D*—H	H···*A*	*D*···*A*	*D*—H···*A*
N1—H1···N2^i^	0.88 (1)	2.22 (2)	3.059 (5)	159 (4)
N1—H2···O2^ii^	0.87 (3)	2.56 (5)	3.324 (4)	147 (4)
C18—H18···*Cg*1^iii^	0.93	2.87	3.528 (4)	129

Symmetry codes: (i) −*x*+1, −*y*+1, −*z*+1; (ii) −*x*, *y*−1/2, −*z*+3/2; (iii) *x*, −*y*+3/2, *z*−1/2.
